# Deciphering the link: ferroptosis and its role in glioma

**DOI:** 10.3389/fimmu.2024.1346585

**Published:** 2024-01-23

**Authors:** He Wang, Yingfeng Liu, Shusheng Che, Xiangjun Li, Dongxue Tang, Shaojing Lv, Hai Zhao

**Affiliations:** ^1^ Department of Neurosurgery, The Affiliated Hospital of Qingdao University, Qingdao, Shandong, China; ^2^ Department of Neurosurgery, Tianshui First People's Hospital, Tianshui, China; ^3^ Department of Breast Surgery, School of Medicine, Qingdao University, Qingdao, Shandong, China; ^4^ Department of Operating Room, The Affiliated Hospital of Qingdao University, Qingdao, Shandong, China

**Keywords:** ferroptosis, glioma, GBM, reactive oxygen species, target therapy

## Abstract

Glioma, as the most frequently occurring primary malignancy in the central nervous system, significantly impacts patients’ quality of life and cognitive abilities. Ferroptosis, a newly discovered form of cell death, is characterized by significant iron accumulation and lipid peroxidation. This process is fundamentally dependent on iron. Various factors inducing ferroptosis can either directly or indirectly influence glutathione peroxidase, leading to reduced antioxidant capabilities and an increase in lipid reactive oxygen species (ROS) within cells, culminating in oxidative cell death. Recent research indicates a strong connection between ferroptosis and a range of pathophysiological conditions, including tumors, neurological disorders, ischemia-reperfusion injuries, kidney damage, and hematological diseases. The regulation of ferroptosis to intervene in the progression of these diseases has emerged as a major area of interest in etiological research and therapy. However, the exact functional alterations and molecular mechanisms underlying ferroptosis remain to be extensively studied. The review firstly explores the intricate relationship between ferroptosis and glioma, highlighting how ferroptosis contributes to glioma pathogenesis and how glioma cells may resist this form of cell death. Then, we discuss recent studies that have identified potential ferroptosis inducers and inhibitors, which could serve as novel therapeutic strategies for glioma. We also examine the current challenges in targeting ferroptosis in glioma treatment, including the complexity of its regulation and the need for precise delivery methods. This review aims to provide a comprehensive overview of the current state of research on ferroptosis in glioma, offering insights into future therapeutic strategies and the broader implications of this novel cell death pathway in cancer biology.

## Introduction

**Figure 1:**
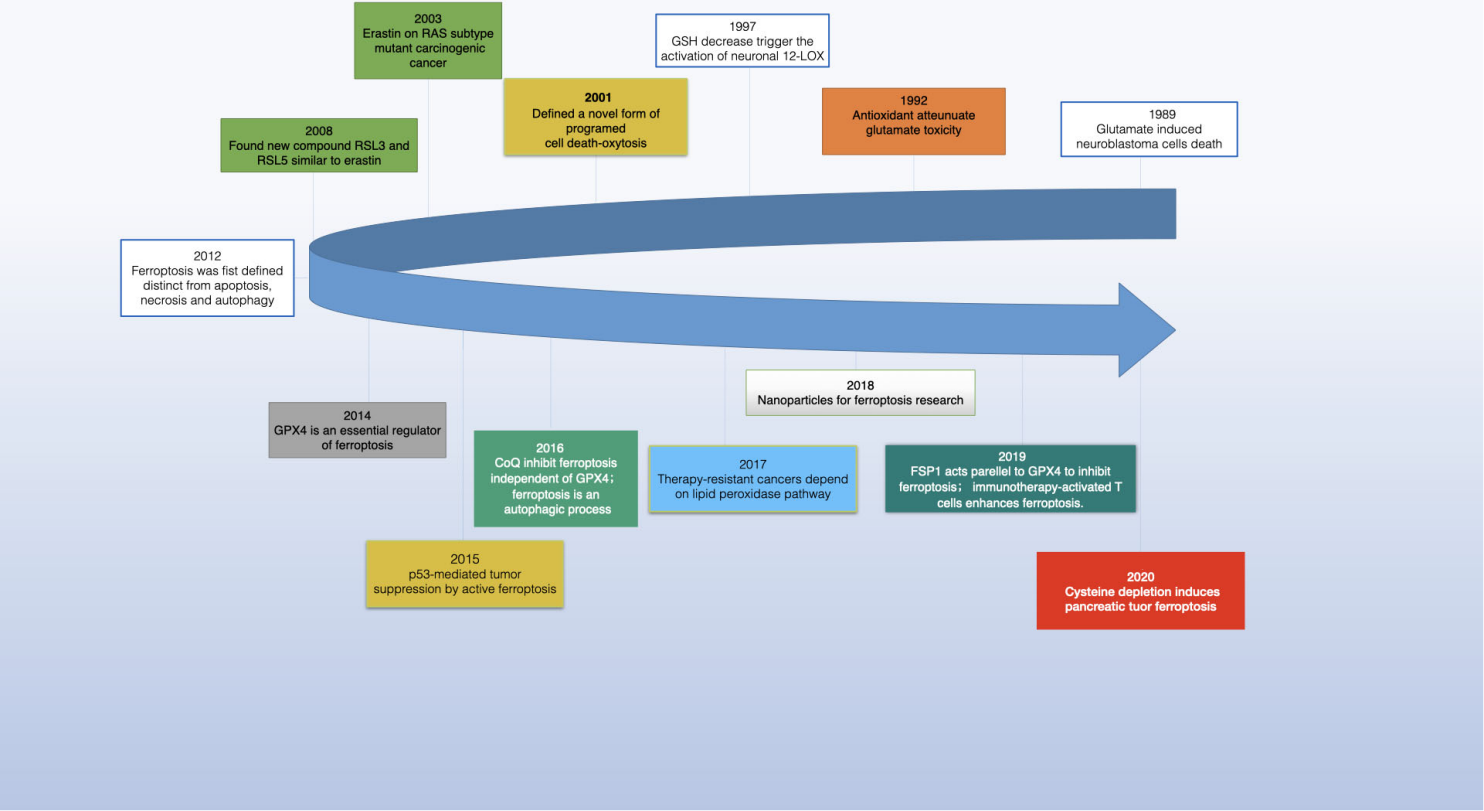
The development of ferroptosis in cancer research.

In 2003, Dolma and team identified a novel compound, erastin, with a unique ability to selectively kill cancer cells expressing the RAS gene, marking a departure from known cell death mechanisms (1). This process lacked traditional signs like nuclear morphological changes, DNA fragmentation, or caspase activation and was unaffected by caspase inhibitors. Further studies by Yang and Yagoda revealed that this type of cell death could be thwarted by iron chelators, and they also discovered RSL3, another compound capable of inducing the same cell death pattern (2, 3). In 2012, Dixon et al. coined the term “ferroptosis” for this form of cell death while investigating how erastin killed RAS-mutant cancer cells (4) (see **Figure 1** *)*. Ferroptosis is characterized morphologically by decreased mitochondrial volume, denser mitochondrial bilayer membranes, and often diminished or absent mitochondrial cristae yet with an intact cell membrane, normal-sized nucleus, and no chromatin condensation (2, 4–6). Biochemically, it involves the depletion of intracellular glutathione (GSH) and a decrease in glutathione peroxidase 4 (GPX4) activity, leading to unmetabolized lipid peroxides (7–10). The presence of Fe2+ causes a Fenton-like oxidation of lipids, resulting in a surge of ROS that promotes ferroptosis (2, 11, 12). Genetically, it’s regulated by multiple genes, primarily those associated with iron homeostasis and lipid peroxidation metabolism, though the specific mechanisms are still under investigation.

**Figure 2:**
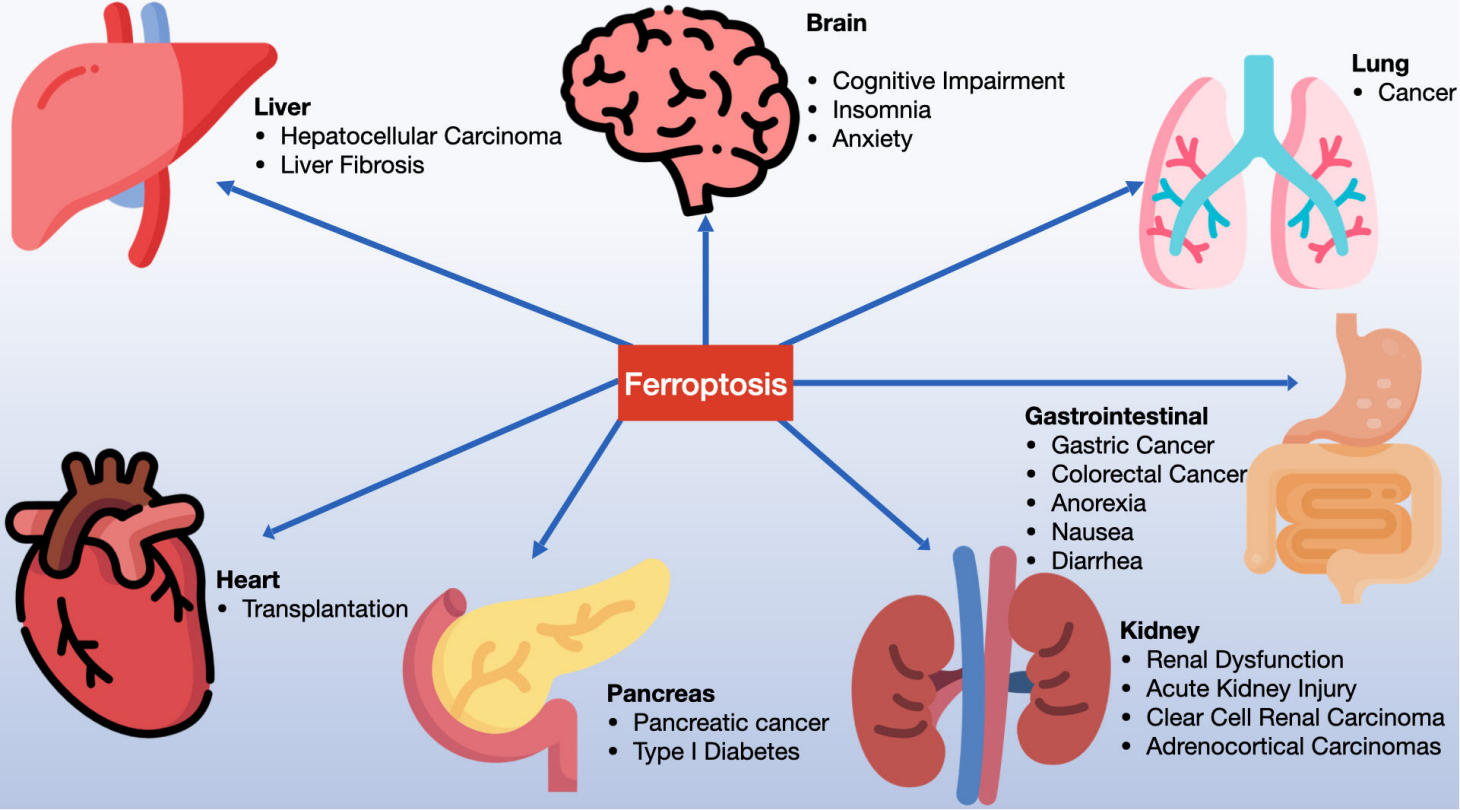
Ferroptosis is significantly involved in a variety of systemic diseases, including disorders of the nervous system, cardiac diseases, hepatic diseases, gastrointestinal conditions, pulmonary diseases, renal diseases, pancreatic disorders, among others.

Ferroptosis inducers are categorized into four groups. The first includes erastin, which reduces GSH levels by inhibiting system Xc- and affects voltage-dependent anion channels (VDACs) for mitochondrial dysfunction. Erastin also enhances lysosomal-associated membrane protein 2a, promoting chaperone-mediated autophagy and GPX4 degradation (13–16). The second category, with members like RSL3 and DPI7, directly inhibits GPX4 (17, 18). The third category features FIN56, which induces ferroptosis by promoting GPX4 degradation and binding to squalene synthase, leading to coenzyme Q10 (COQ10) depletion and enhanced cell sensitivity to ferroptosis (19–21). Lastly, FINO2, resembling artemisinin, causes ferroptosis through labile iron oxidation and GPX4 inactivation (21–23). Research has also unearthed specific ferroptosis inhibitors like ferrostatin-1 (Fer-1), liproxstatin-1, and vitamin E, along with iron chelators, which prevent lipid peroxide formation. In 2014, Skouta et al. found that Fer-1 inhibited cell death in models of Huntington’s disease (HD), periventricular white matter (PVL), and renal insufficiency, highlighting the potential of ferrostatin in disease models and emphasizing the significance of ferroptosis beyond cell culture (24). The discovery of ferroptosis thus opens new avenues for understanding and treating various diseases (see **Figure 2** *)*.

Glioma, the most prevalent primary brain tumor, represents about 50-60% of central nervous system (CNS) tumors and roughly 81%’, of all intracranial malignancies (25, 26). Compared to other CNS tumors, gliomas exhibit notably higher recurrence rates. The World Health Organization (WHO) grades gliomas from 1 to 4, with grades 1 and 2 being low-grade and grades 3 and 4 as high-grade gliomas (27). The median overall survival (OS) for patients with low-grade gliomas is about 11.6 years, but this significantly drops to around three years for grade 3 gliomas and approximately 15 months for those with grade 4 gliomas (28, 29). Despite current treatments like surgical resection, radiotherapy, chemotherapy, novel molecular targeted therapies, and immunotherapy, patient outcomes remain largely unsatisfactory, with a dire prognosis. This underscores the urgent need for new therapeutic approaches and targets to enhance OS and life quality in glioma patients. Traditionally, cell death includes necrosis, apoptosis, autophagy, and pyroptosis (30). However, the recent focus has shifted to ferroptosis, a novel non-apoptotic form of cell death caused by iron-dependent lipid peroxidation, garnering increased research interest (4, 31, 32). In this review, we will primarily concentrate on the key molecular processes of ferroptosis and its possible effects on the development and treatment of gliomas, especially GBM. Additionally, we will offer a summary of the difficulties associated with using ferroptosis in glioma treatment and explore the therapeutic possibilities of leveraging ferroptosis to enhance treatment outcomes.

## Discovery and characteristics of ferroptosis

The concept of “ferroptosis,” first introduced by Dixon and others in 2012, was preceded by a similar type of cell death termed “oxytosis” identified by Murphy and colleagues in 1989 (33). Oxytosis, caused by cysteine depletion in neurons, was linked to the inhibition of SLC7A11, a component crucial in ferroptosis. Both oxytosis and ferroptosis share similarities like gene expression patterns, lipoxygenase activity, and reactive oxygen species accumulation (34). By 2003, a unique form of cell death in RAS-expressing cancer cells, not prevented by caspase inhibitors but mitigated by iron-chelating agents, was observed (1, 3). This led to the discovery that RSL3, a RAS-selective lethal small molecule, could also induce this iron-dependent cell death, which Dixon et al. named ferroptosis (2).

Morphologically, ferroptosis is marked by mitochondrial changes such as shrinkage and decreased cristae. Biochemically, it involves oxidative stress and reduced antioxidative defense. The exact mechanisms, particularly how phospholipid peroxidation triggers ferroptosis and its specific epilipidomic patterns in different tissues and diseases, are still being researched. Emerging research has begun to reveal key regulators of ferroptosis. For instance, the glutathione/glutathione peroxidase 4 pathway, ACSL4, and selenium’s protective role against ferroptosis have been identified. The FSP1-CoQ10-NAD(P)H pathway and the GCH1-BH4 pathway were also recognized as GPX4-independent mechanisms influencing ferroptosis (35, 36). Recently, the DHODH-CoQ10 axis in the mitochondrial inner membrane and vitamin K’s role as an FSP1-dependent ferroptosis inhibitor were discovered, highlighting the expanding understanding of this cellular process (37).

## Molecular mechanisms of ferroptosis

In 1984, J.M. Gutteridge discovered that iron salts can initiate lipid peroxidation by converting lipid peroxides into alkoxyl and peroxyl radicals (38). He also found that iron, when combined with ethylene diamine tetraacetic acid (EDTA), can start lipid peroxidation by reacting with hydrogen peroxide (H2O2) to create hydroxyl radicals (•OH) (38). This research laid the groundwork for understanding iron-dependent cell death. Additionally, it was observed that external glutamate can trigger cell death by inhibiting cystine uptake via xCT, leading to reduced glutathione production. This process, known as oxytosis, is reliant on oxidative stress and reactive oxygen species (ROS) production and set the stage for the concept of ferroptosis.

In 2012, Brent R. Stockwell defined ferroptosis as a new form of programmed cell death, specifically emphasizing its role in iron-dependent cell death in cancer cells. The molecular mechanisms of ferroptosis are distinct from other well-known forms of regulated cell death (RCD). Key biochemical events in ferroptosis include the accumulation of excess iron and ROS in cells, extensive lipid peroxidation, inactivation of xCT, and the depletion of glutathione and lipid repair enzymes. This understanding marks a significant advancement in the study of cell death and its implications in diseases like cancer (39–41).

### Iron metabolism

Iron plays a crucial role in regulating ferroptosis, not only by triggering the non-enzymatic Fenton reaction that directly oxidizes polyunsaturated fatty acid-phospholipids (PUFA-PLs), but also by serving as a vital cofactor for enzymes involved in lipid peroxidation, such as ALOX and POR (42–45). Under normal circumstances, cells maintain a relatively stable level of labile iron through a finely tuned balance of iron absorption, use, storage, and export. However, disruptions in these iron metabolism processes can either accelerate or inhibit ferroptosis, depending on whether they increase or decrease the intracellular labile iron pool. For instance, most intracellular iron is stored as inert iron within ferritin. The autophagic breakdown of ferritin, known as ferritinophagy, releases iron from ferritin into the labile iron pool. Blocking NCOA4-mediated ferritinophagy lowers the labile iron pool and thus inhibits ferroptosis (46, 47). On the other hand, promoting ferritinophagy, such as by inhibiting cytosolic glutamate oxaloacetate transaminase 1 (GOT1), increases the labile iron pool and encourages ferroptosis. The exact mechanism by which GOT1 inhibition facilitates the release of labile iron through ferritinophagy is still under investigation (48–50). For a more comprehensive discussion on iron metabolism in the context of ferroptosis, interested readers are directed to recent detailed reviews on the subject (51).

**Figure 3:**
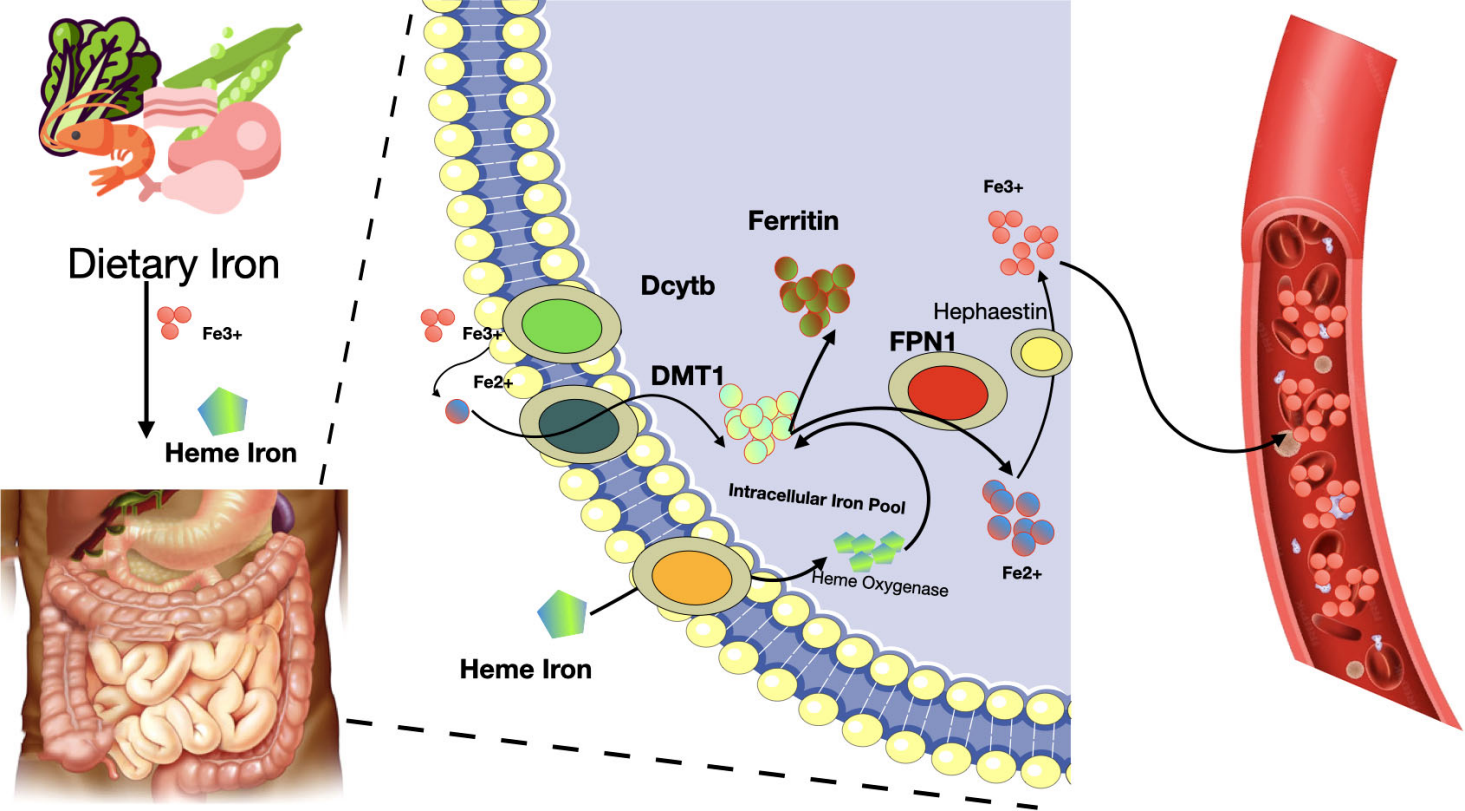
Iron absorption and metabolism in the body. Fe2+, ferrous cation; Fe3+, ferric cation; Dcytb, duodenal cytochrome b; DMT1, divalent metal transporter 1; FPN1, ferroportin 1.

Iron absorption and metabolism in the body can be summarized as following. Firstly, iron is a crucial trace element necessary for various biological functions in cells. Dietary iron is mainly categorized into non-heme and heme iron, with the latter being Fe2+ combined with protoporphyrin IX. Non-heme iron primarily exists as ferric salts in the diet, which are converted to Fe2+ by intestinal iron reductase. This Fe2+ is then absorbed by intestinal epithelial cells (IECs) through the DMT1 transporter and exits these cells via ferroportin 1 (FPN1) (52, 53). Once in the bloodstream, Fe2+ is oxidized to Fe3+ by ceruloplasmin (CP) and hephaestin (HP), and then binds to transferrin (Tf) for transport (54). Tf-Fe3+ attaches to cell membranes via the transferrin receptor (TfR), internalizes into cells as endosomes, and is reduced back to Fe2+ by STEAP3 in various cells before entering the cytoplasm through DMT1 on the endosomal membrane (55) (see **Figure 3** *)*.

Heme iron, a component of hemoglobin and myoglobin, has a less clear absorption mechanism. It’s believed that heme iron ingested is broken down by heme oxygenase in intestinal cells, releasing free ferric iron. Subsequently, a significant amount of Fe2+ accumulates in the cytoplasm, forming a labile iron pool crucial for processes like ferroptosis (56, 57). Excessive intracellular iron, in the presence of H2O2, triggers the Fenton reaction, leading to ROS formation such as •OH, which induces lipid peroxidation and hence ferroptosis (58–60). Iron responsive element binding protein 2 (IREB2) is an important regulator of iron metabolism and may influence sensitivity to ferroptosis (61–63). Autophagy also plays a role in iron regulation, affecting the recruitment of ferritin to autophagosomes for lysosomal degradation, thus releasing free iron (64–66). For example, ferritinophagy, facilitated by the cargo receptor NCOA4, specifically targets the ferritin heavy chain 1 (FTH1), releasing iron into autophagosomes for degradation (67). Conversely, a decrease in intracellular Fe2+ levels can hinder ferroptosis, as seen when erastin-induced ferroptosis is reduced following the knockout of autophagy-related genes ATG5 or ATG7 (68, 69). Therefore, iron metabolism is integral to the process of ferroptosis.

### Lipid metabolism

**Figure 4:**
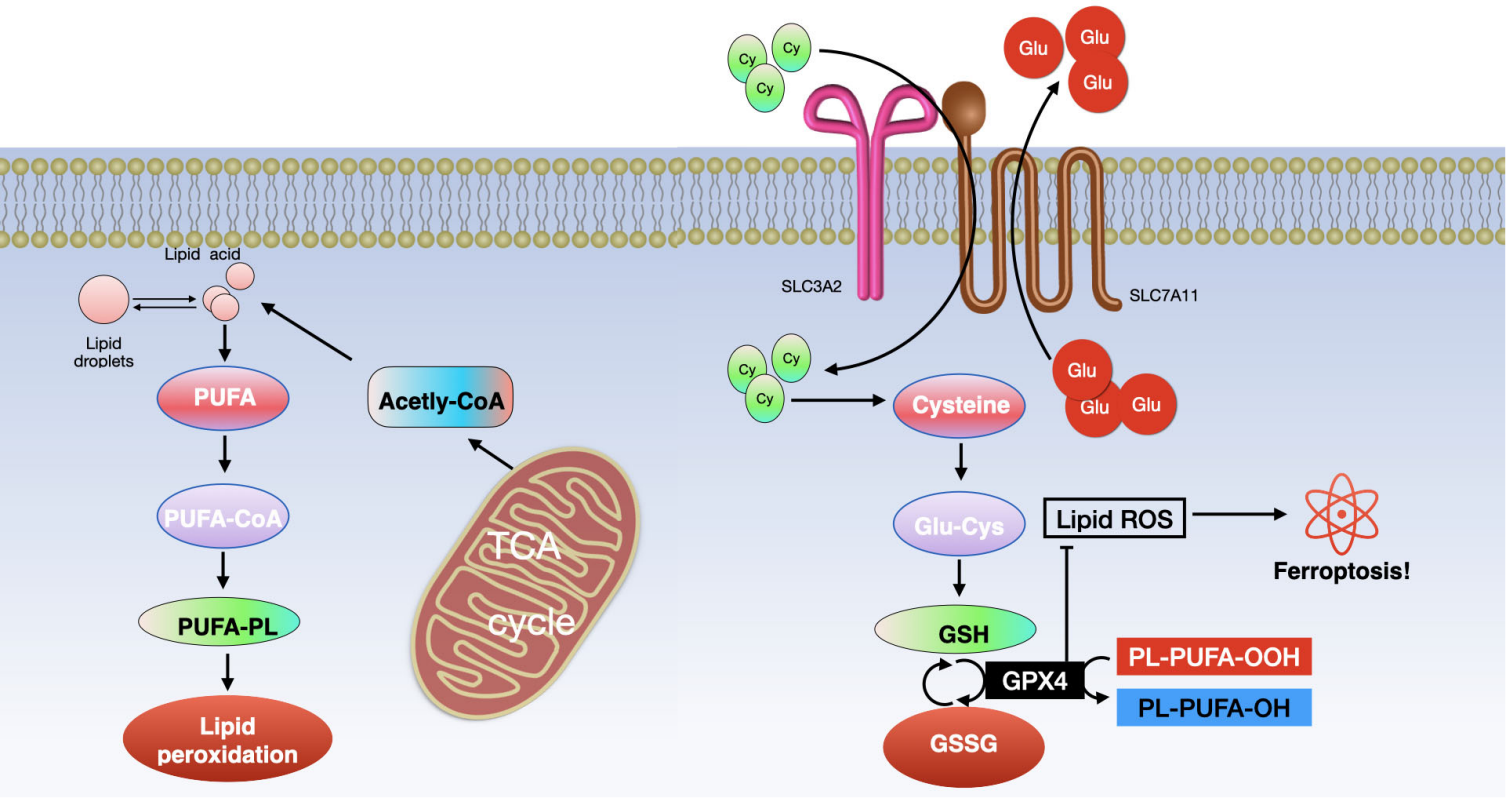
Ferroptosis involves two primary molecular pathways. (Left) The pathway of lipid metabolism and (Right) the xCT/GPX4 pathway. Key components include ACSL4 (long-chain fatty acid CoA ligase 4), PUFA (polyunsaturated fatty acid), LPCAT3 (lysophosphatidylcholine acyltransferase 3), PUFA-CoA (polyunsaturated fatty acid coenzyme A), ALOXs (lipoxygenases), PUFA-PL (phospholipid containing polyunsaturated fatty acid), TCA (tricarboxylic acid cycle), GSH (glutathione), GSSG (glutathione disulfide), and GPX4 (glutathione peroxidase 4).

Lipid peroxidation stands out as a key characteristic and the primary mechanism that drives ferroptosis. Reactive oxygen species (ROS) generated through the Fenton reaction interact with polyunsaturated fatty acids (PUFAs) in cell or organelle membranes, resulting in the formation of toxic phospholipid hydroperoxides (PLOOHs) and triggering ferroptosis (70–72). Various factors contribute to the process of lipid peroxidation, including enzymes such as acyl–coenzyme A synthetase long-chain family member 4 (ACSL4), lysophosphatidylcholine acyltransferase 3 (LPCAT3), and lipoxygenases (LOXs) (73–75). ACSL4, vital for lipid metabolism, and LPCAT3, key in catalyzing the reacylation of lysophospholipids to phospholipids, play crucial roles (76–78). They activate free long-chain PUFAs, facilitate the conversion of lysophosphatidylcholine (LPC) to lecithin, mediate the synthesis of oxidized phospholipids in cell membranes, and thus influence ferroptosis. ACSL4 specifically esterifies arachidonic acid (AA) into acyl-coenzyme A (acyl-CoA), essential for PUFA biosynthesis, which is pivotal in lipid peroxidation and ferroptosis. LOXs are critical regulators in this process. They significantly impact the onset of ferroptosis by promoting lipid autoxidation and are indicative of a cell’s sensitivity to ferroptosis (74, 79). Therefore, in the context of ferroptosis, lipid peroxidation leads to cell death by disrupting the lipid bilayer structure of cellular and organelle membranes (see **Figure 4** *)*.

### Mitochondrial metabolism

Mitochondrial metabolic processes play a crucial role in initiating ferroptosis (80). One key aspect is the generation of mitochondrial reactive oxygen species (ROS), essential for lipid peroxidation and the onset of ferroptosis. Mitochondria are significant cellular sources of ROS, where electron leakage from complexes I and III of the electron transport chain leads to the formation of superoxides. These superoxides are then converted into hydrogen peroxide (H2O2) by superoxide dismutase (81). The H2O2 reacts with labile iron through the Fenton reaction to produce hydroxyl radicals, which in turn drive the peroxidation of polyunsaturated fatty acid-phospholipids (PUFA-PLs) (81, 82). Additionally, mitochondrial electron transport and proton pumping, crucial for ATP production, also play a role in promoting ferroptosis (83–86). Under conditions where ATP is depleted, AMP-activated protein kinase (AMPK) phosphorylates and inhibits acetyl-CoA carboxylase (ACC), leading to a suppression of PUFA-PL synthesis and thus blocking ferroptosis. Conversely, when ATP levels are sufficient, AMPK activation is inefficient, resulting in the activation of ACC and promotion of PUFA-PL synthesis and ferroptosis (85, 86). Furthermore, mitochondria’s role in biosynthetic pathways, such as the tricarboxylic acid (TCA) cycle and anaplerotic reactions (like glutaminolysis), contributes to ferroptosis (83). These processes likely drive ferroptosis through the promotion of ROS, ATP, and/or PUFA-PL generation (87–89). Hence, the multifaceted functions of mitochondria in bioenergetics, biosynthesis, and ROS generation are central to driving mitochondrial lipid peroxidation and ferroptosis (80).

### The xCT and GPX4

Environmental stresses like high temperature and hypoxia can trigger iron reactions, necessitating cells to establish defense mechanisms against ferroptosis. The most classic defense against ferroptosis involves the antioxidant axis consisting of xCT, glutathione (GSH), and GPX4. xCT, a transmembrane protein, comprises the light-chain solute carrier family 7 member 11 (SLC7A11) and the heavy-chain solute carrier family 3 member 2 (SLC3A2, also known as CD98hc or 4F2hc) (90, 91). SLC7A11, the primary functional unit of xCT, regulates the intake of extracellular cysteine (Cys) into cells and the export of intracellular glutamic acid (Glu). SLC3A2 helps maintain xCT’s stability by anchoring and stabilizing SLC7A11 (92).

Cysteine is converted into reduced GSH alongside Glu and glycine (Gly) under the influence of glutamate cysteine ligase (GCL) and glutamylcysteine synthetase (GCS) (93, 94). Beclin 1 can suppress xCT activity, thereby promoting ferroptosis, by directly binding to SLC7A11 (95). GPX4, a crucial enzyme in preventing ferroptosis, reduces toxic phospholipid hydroperoxides (PLOOH) to non-toxic phospholipid alcohols (PLOH) in membranes, using GSH (96). The inhibitor 2-cyano-3,12-dioxooleana-1,9 (11)-dien-28-oic acid (CDDO) helps prevent the specific degradation of GPX4 through chaperone-mediated autophagy (CMA). It does so by affecting the interaction between heat shock protein 90 (HSP90) and lysosomes, thus inhibiting cell ferroptosis (13). However, this inhibition can be counteracted by suppressing the mammalian target of rapamycin (MTOR) pathway, which may lead to GPX4 degradation and promote ferroptosis (97). Consequently, xCT, GPX4, and GSH are key regulators and have a significant impact on the control of ferroptosis (see **Figure 4**).

### FSP1-CoQH2 system

The FSP1-CoQH2 system has challenged the earlier belief that GPX4 was the sole defense mechanism against ferroptosis. Recent studies have shown that ferroptosis suppressor protein 1 (FSP1, also known as AIFM2) works independently of GPX4 to protect against ferroptosis (98, 99). FSP1, located on the plasma membrane and other subcellular compartments, is crucial in this role. Its presence on the plasma membrane is both necessary and sufficient for its function in ferroptosis suppression (98, 99). FSP1 acts as an NAD(P)H-dependent oxidoreductase, reducing ubiquinone (also known as coenzyme Q or CoQ) to ubiquinol (CoQH2). CoQH2, apart from its known role in mitochondrial electron transport, can intercept lipid peroxyl radicals, thus inhibiting lipid peroxidation and ferroptosis (98, 99). The generation of a non-mitochondrial CoQH2 pool by FSP1 as radical-trapping antioxidants plays a key role in its anti-ferroptosis activity (98, 99). Though CoQ is mainly produced in mitochondria, it is also found in non-mitochondrial membranes, including the plasma membrane (100–102). The exact sources of non-mitochondrial CoQ used by FSP1 in ferroptosis defense are yet to be determined.

### DHODH-CoQH2 system

Additionally, the DHODH-CoQH2 system has been identified as another mitochondria-localized defense mechanism. This system, mediated by dihydroorotate dehydrogenase (DHODH), can compensate for the loss of GPX4 in detoxifying mitochondrial lipid peroxidation (36). DHODH, an enzyme in pyrimidine synthesis, reduces CoQ to CoQH2 in the inner mitochondrial membrane (36). When GPX4 is acutely inactivated, DHODH activity increases, leading to more CoQH2 production which neutralizes lipid peroxidation and protects against mitochondrial ferroptosis. Inactivating both mitochondrial GPX4 and DHODH leads to significant mitochondrial lipid peroxidation and ferroptosis (36). Notably, while mitochondrial GPX4 and DHODH can substitute for each other in reducing mitochondrial lipid peroxidation, cytosolic GPX4 and FSP1 cannot, likely due to their non-mitochondrial localization.

These findings suggest a model where ferroptosis defense systems are divided into two main categories: the GPX4 system and the CoQH2 system, each further divided into non-mitochondrial and mitochondrial components. For instance, cytosolic and mitochondrial GPX4 are part of the GPX4 system, while non-mitochondrial FSP1 and mitochondrial DHODH belong to the CoQH2 system. This division is presumably due to the need to mitigate lipid peroxides in mitochondria and the unique double-membrane structure of mitochondria, which limits the effectiveness of defense systems located in other compartments (103). However, further research is required to fully understand this compartmentalization model in ferroptosis regulation and reconcile it with some conflicting findings, such as the significant localization of cytosolic GPX4 in the intermembrane space of mitochondria (104). The role of cytosolic GPX4 in mitochondria and its potential impact on suppressing mitochondrial lipid peroxidation needs further investigation.

### GCH1–BH4 system

Recent research has highlighted the role of GTP cyclohydrolase 1 (GCH1) as a key player in regulating ferroptosis (105, 106). GCH1 is involved in the rate-limiting step of the biosynthesis pathway for tetrahydrobiopterin (BH4), a cofactor for aromatic amino acid hydroxylases and other enzymes (107). BH4, aside from its role as a cofactor, is a radical-trapping antioxidant capable of capturing lipid peroxyl radicals, thus contributing to the inhibition of ferroptosis independently of its cofactor function (106). It is suggested that GCH1 helps prevent ferroptosis in two ways: firstly, through the generation of BH4 as a radical-trapping antioxidant, and secondly, via the GCH1-mediated production of coenzyme QH2 (CoQH2) and phospholipids (PLs) containing two polyunsaturated fatty acid (PUFA) tails. This is notable because most PUFA-PLs typically exhibit an asymmetric structure, with a PUFA tail at the sn-2 position and a saturated fatty acid tail at the sn-1 position (105, 106). The specific subcellular location where the GCH1-BH4 system functions, however, is still to be determined. This discovery adds another layer to the complex regulatory network of ferroptosis and underscores the multifaceted nature of cellular defense mechanisms against this form of cell death.

## Ferroptosis in the tumor microenvironment

Recent studies have revealed the significant role of the tumor microenvironment (TME), particularly its immune cells, in determining the occurrence of tumor-cell ferroptosis (108). CD8+ cytotoxic T cells, key players in antitumor immunity within the TME, release interferon-γ (IFNγ), which inhibits cystine uptake in cancer cells by downregulating SLC7A11 expression (109). This action enhances lipid peroxidation and ferroptosis in tumors. Intriguingly, IFNγ also suppresses SLC7A11-mediated cystine transport in macrophages, indicating that IFNγ can regulate SLC7A11 in both cancer and non-cancer contexts. Furthermore, combining immune checkpoint inhibitors (ICIs) with cyst(e)inase intensifies T cell-mediated antitumor responses by synergistically promoting tumor ferroptosis. This suggests that ferroptosis plays a crucial role in T cell-mediated antitumor activity, and targeting SLC7A11 in conjunction with ICIs could be a promising cancer treatment strategy (108).

Ferroptotic cancer cells release various immunostimulatory signals like high mobility group box 1 (HMGB1), calreticulin, ATP, and phosphatidylethanolamine (110–112). These signals aid dendritic cell maturation, enhance macrophage phagocytosis of ferroptotic cancer cells, and boost the infiltration of CD8+ T cells into tumors. Early ferroptotic cells, after short-term treatment with a GPX4 inhibitor, can induce dendritic cell maturation and activate antitumor immunity, similar to a vaccination effect (111). This supports the notion of ferroptosis as a form of immunogenic cell death.

**Figure 5:**
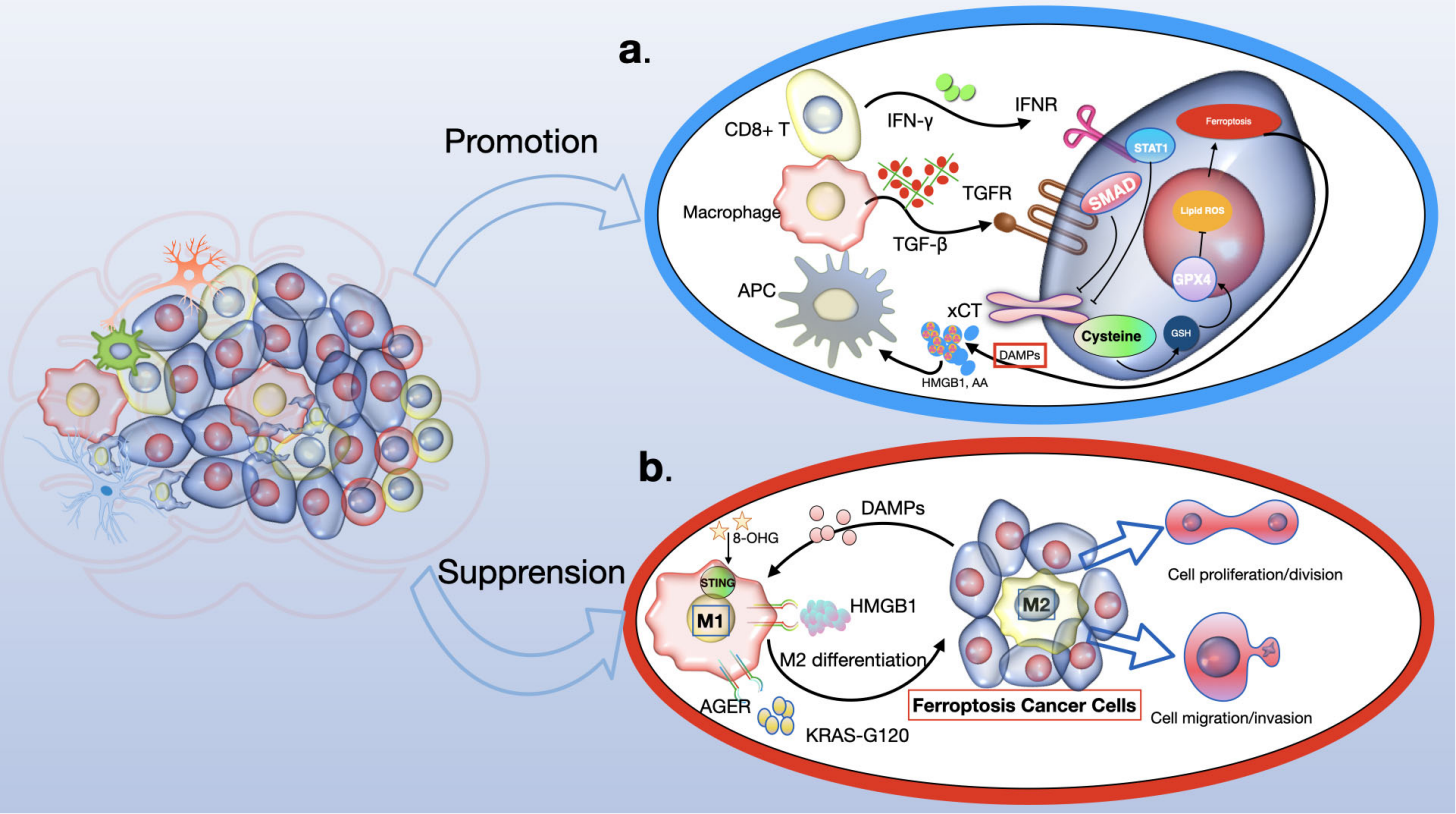
The role of ferroptosis in anti-tumour immunity. Ferroptosis has a dual role to play in antitumour immunity, dependent on the nature of the immune cell. **(A)** In the context of tumor immunity, CD8+ T cells secrete IFNγ, which activates the INFR pathway, leading to the suppression of SLC7A11 expression via STAT1. This process enhances ferroptosis in cancer cells. Additionally, macrophage-derived TGFβ1 suppresses the xCT system through SMAD proteins, further inducing lipid ROS-dependent ferroptosis through the GSH-GPX4 pathway. Consequently, ferroptotic glioma cells emit DAMPs (like HMGB1 and AA), which encourage the attraction and activation of immune cells. **(B)** Conversely, DAMPs such as HMGB1, KRAS-G12D, and 8-OHG influence the behavior of macrophages within the tumor milieu. Specifically, KRAS-G12D interacts with AGER on macrophage surfaces, leading to M2 macrophage polarization. This could potentially impede the effectiveness of the immune system's response against the tumor. IFN-γ, interferon-γ; INFR, interferon receptor; STAT1, signal transducer and activator of transcription 1; GSH, glutathione; GPX4, glutathione peroxidase 4; DAMPs, damage-associated molecular patterns; HMGB1, high mobility group protein B1; AA, arachidonic acid; 8-OHG, 8-hydroxyguanosine; AGER, advanced glycosylation end product-specific receptor.

Ferroptosis induction in certain immunosuppressive cells also enhances antitumor immunity. Regulatory T (Treg) cells, which usually suppress tumor surveillance, are resistant to ferroptosis due to GPX4 induction (113, 114). However, inducing ferroptosis in Treg cells through Gpx4 deletion contributes to antitumor immunity. Similarly, targeting myeloid-derived suppressor cells (MDSCs) for ferroptosis, by inhibiting ASAH2-mediated suppression of the p53–haeme oxygenase 1 (HMOX1) axis, activates tumor-infiltrating cytotoxic CD8+ T cells and suppresses tumors (114, 115). Tumor-associated macrophages (TAMs) that exhibit an M2-like phenotype and suppress antitumor immunity are more vulnerable to ferroptosis induced by GPX4 inhibition than M1-like TAMs, which promote antitumor immunity (116). Inducing ferroptosis in M2-like TAMs without affecting M1-like TAMs could overcome the immunosuppressive TME and enhance cancer immunotherapy (117, 118) (see **Figure 5**).

However, evidence also suggests that ferroptosis can promote tumor growth in the context of tumor immunity. Gpx4-deficient T cells from specific knockout mice rapidly accumulate lipid peroxides upon activation, leading to ferroptosis (119). CD8+ T cells from tumors, but not from lymph nodes, show substantial lipid peroxide accumulation, indicating a vulnerability to ferroptosis. This could weaken antitumor immunity and aid tumor growth (120). CD36, which mediates fatty acid uptake in tumor-infiltrating CD8+ T cells, induces lipid peroxidation and ferroptosis, compromising antitumor immunity. Blocking CD36 or ferroptosis in CD8+ T cells restores their antitumor activity, while treating them with GPX4 inhibitors leads to impaired antitumor effects (121). Furthermore, T follicular helper (TFH) cells, a CD4+ T cell subset favoring antitumor immunity, are highly susceptible to ferroptosis, with GPX4 being crucial for their survival and function (122). The synergistic enhancement of antitumor immunity by targeting SLC7A11 along with ICIs may be due to the limited effect of Slc7a11 knockout or cystine deprivation on T cell viability or antitumor effects, possibly because of SLC7A11’s low expression and non-essential role in T cells (123). The contrasting effects of GPX4 deletion versus SLC7A11 deletion on T cell function are not fully understood but might be related to SLC7A11’s limited role in these cells (124, 125).

## Therapeutic strategies targeting ferroptosis

### Small molecules to induce ferroptosis in cancer

System Xc− plays a significant role in the survival and growth of many tumor cells, making it a potential target for cancer treatment. Erastin, known for inhibiting system Xc−, reduces glutathione (GSH) levels and induces ferroptosis. A notable discovery in Dixon’s 2012 study was that erastin led to ROS accumulation in NRAS-mutant HT-1080 fibrosarcoma cells, and this cell death was hindered by the iron chelator deferoxamine, indicating erastin’s role in inducing ferroptosis (4, 96, 126, 127). Further research highlighted the importance of the RAF/MEK/ERK signaling pathway in erastin-triggered ferroptosis in RAS-mutated cancers (128). Derivatives of erastin, like piperazine erastin and imidazole ketone erastin (IKE), have been developed to address erastin’s limitations, such as poor water solubility and unstable *in-vivo* metabolism. For instance, IKE has been effectively used in treating diffuse large B cell lymphoma (DLBCL) in the SUDHL6 xenograft animal model (129).

Sorafenib, a multi-kinase inhibitor used in treating advanced renal cell carcinoma, thyroid carcinoma, and hepatocellular carcinoma, is another inducer of ferroptosis. Its cytotoxicity to hepatocellular carcinoma was negated when treated with an iron chelator (130). However, some cancer cell lines have developed resistance to sorafenib, as seen in hepatocellular carcinoma cells with retinoblastoma (Rb) protein, where sorafenib-induced ferroptosis was inhibited (131). Additionally, the anti-inflammatory drug sulfasalazine (SAS) can induce ferroptosis in glioma cells by inhibiting system Xc− (132).

**Figure 6:**
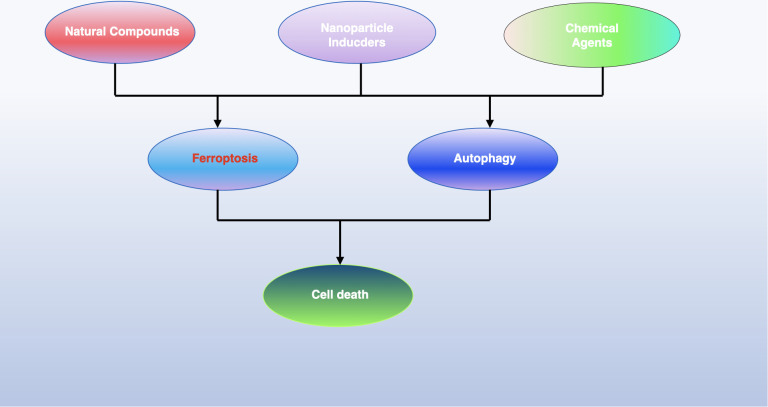
The iron maiden. Autophagy is an essential, ancient mechanism that preserves balance within cells by eliminating harmful components. It serves a dual function in determining cell fate, being implicated in both survival and death processes. Recent findings have shown that autophagy contributes to the initiation of ferroptosis, an iron-dependent form of cell death characterized by the build-up of toxic lipid peroxides. This review sheds light on the interaction between autophagy and ferroptosis and proposes that understanding this relationship could open new avenues for cancer treatment strategies.

Some cancer cells induce ferroptosis through the transsulfuration pathway instead of system Xc−. GPX4 inactivation can eliminate these tumor cells, as seen with (1S, 3R)-RSL, which induces ferroptosis through direct GPX4 inhibition, and FIN56, which promotes ferroptosis by degrading GPX4 (133). Ferroptosis can also be induced by increasing the labile iron pool (LIP). Compounds like BAY 11-7085 can induce ferroptosis through the Nrf2-SLC7A11-HO-1 pathway, and overexpression of heme oxygenase-1 (HO-1), encoded by HMOX1, has been observed in MDA-MB-231 breast cancer and DBTRG-05MG glioblastoma cells (134). Increased TF expression and decreased FPN-1 expression, mediated by compounds like siramesine and lapatinib, can also induce ferroptosis. Autophagy contributes to this process by degrading ferritin in cancer cells. The cargo receptor nuclear receptor coactivator 4 (NCOA4) is significant in the autophagic turnover of ferritin in ferroptosis. In pancreatic cancer cells, NCOA4 overexpression inhibited FIH1 expression and promoted erastin-induced ferroptosis (47). Further research is necessary to discover novel molecules targeting NCOA4 for cancer treatment through ferroptosis. Besides these, more small molecules are being studied for their potential to induce ferroptosis in cancer therapy (see **Figure 6**).

### Nanoparticle inducers of ferroptosis in cancer

Nanotechnology applications, particularly due to their unique physicochemical properties, have garnered significant interest in recent years. Many nanomaterials, including iron-containing nanoparticles, leverage the Fenton reaction for their functionality. For instance, Chen and colleagues developed a tumor-targeted nanoparticle called α‐enolase targeting peptide modified Pt-prodrug loaded Fe3O4 nanoparticles (ETP-PtFeNP). Treatment of tumor cells with ETP-PtFeNP resulted in increased ROS generation, enhanced immunogenicity, and a robust anti-tumor immune response (135). Additionally, a novel nanoparticle named SRF@FeIIITA (SFT) has been shown to be effective in inhibiting tumor progression. This nanoparticle combines photodynamic therapy (PDT) and ferroptosis by loading methylene blue (MB) into SFT. This is achieved by depositing tannic acid (TA) and Fe3+ onto SRF nanocrystals, enabling a dual-therapy approach (136).

Nanomaterials can also trigger ferroptosis through the manipulation of GSH metabolism. Arginine-capped manganese silicate nanobubbles (AMSNs), for example, have been developed to efficiently deplete GSH (137). This efficiency is attributed to their high surface area to volume ratio. *In-vivo* studies have demonstrated that AMSN treatment can suppress the growth of Huh7 xenograft tumors by downregulating GPX4. The effectiveness of AMSN-induced ferroptosis can be inhibited by the ferroptosis inhibitor liproxstatin-1, highlighting the potential of these nanomaterials in targeted cancer therapy through the induction of ferroptosis.

### Ferroptosis modulation for tumor sensitization to anticancer therapies

Drug resistance presents a significant hurdle in chemotherapy treatment, but the use of ferroptosis inducers might offer a way to surmount this challenge. Incorporating ferroptosis agonists with chemotherapy drugs could emerge as an innovative approach to cancer therapy (137). Persister cells, which are cancer cells that survive after multiple rounds of chemotherapy, exhibit downregulated Nrf2-targeted genes (138). Accelerating ferroptosis in these cells can be achieved by inhibiting intracellular NF2 and the hippo signaling pathway (139). Moreover, persister cells typically show reduced levels of glutathione (GSH) and nicotinamide adenine dinucleotide phosphate (NADPH), making them more susceptible to lipid peroxidation. GPX4 inhibitors have been found to be particularly effective against these cells. Therefore, inducing ferroptosis in persister cells may be a promising strategy to overcome their drug resistance, offering a new avenue in the fight against cancer.

### Ferroptosis-associated antitumor combination therapy

There have been limited studies on combining ferroptosis inducers with other anti-tumor therapies for clinical treatment, with many investigations still in the experimental phase. An important aspect in cancer cells, especially when the oxygen concentration is above 3–8% as found in most tissues, is the iron-sulfur cluster biosynthetic enzyme NFS1. Its high expression is often observed in well-differentiated adenocarcinomas. Research by Alvarez SW’s group suggested that inhibiting NFS1, along with suppressing cysteine transport, could induce ferroptosis in tumor cells (140). Interestingly, some tumors that are resistant to certain chemotherapy drugs show a heightened sensitivity to ferroptosis inducers. For instance, pancreatic cancer cells, known for their resistance to chemotherapy-induced apoptosis, demonstrate considerable sensitivity to artemisinin-induced ferroptosis. Thus, ferroptosis inducers emerge as a promising strategy for cancer therapies, particularly for types of cancer that are resistant to conventional treatments.

### Targeting ferroptosis to prevent tumor metastasis

In terms of preventing tumor metastasis, targeting ferroptosis could be a potential approach. Clinical treatment of tumor metastasis is complex due to factors like tumor heterogeneity, oncogene activity, epithelial-mesenchymal transition (EMT), and the microenvironment of metastatic sites (141). Since cancer metastasis can be inhibited by high intracellular oxidative stress, ferroptosis, which involves the accumulation of such stress, may be an effective strategy. Nanoparticles present a substantial advantage in treating cancer metastasis due to their relatively low risk compared to locally injected agents (142). For example, a metal–organic network encapsulating the p53 plasmid (MON-p53) was developed using coordination between ferric iron (Fe3+) and tannic acid (TA). Treatment with MON-p53 showed the potential to suppress cancer cell migration in *in-vitro* wound healing assays, suggesting its possible role in inhibiting tumor metastasis (143, 144). Moreover, mesenchymal cancer cells, known for their metastatic potential and resistance to anti-cancer treatments, could be rendered more sensitive to chemotherapy and thus reduce metastasis. Antagonizing the NF2-YAP pathway, which promotes ferroptosis by up-regulating modulators like ACSL4 and transferrin receptor (TFRC), offers a new perspective on treating mesenchymal or metastatic cancer cells, which are highly sensitive to ferroptosis (139).

## Challenges of ferroptosis in glioma

**Figure 7:**
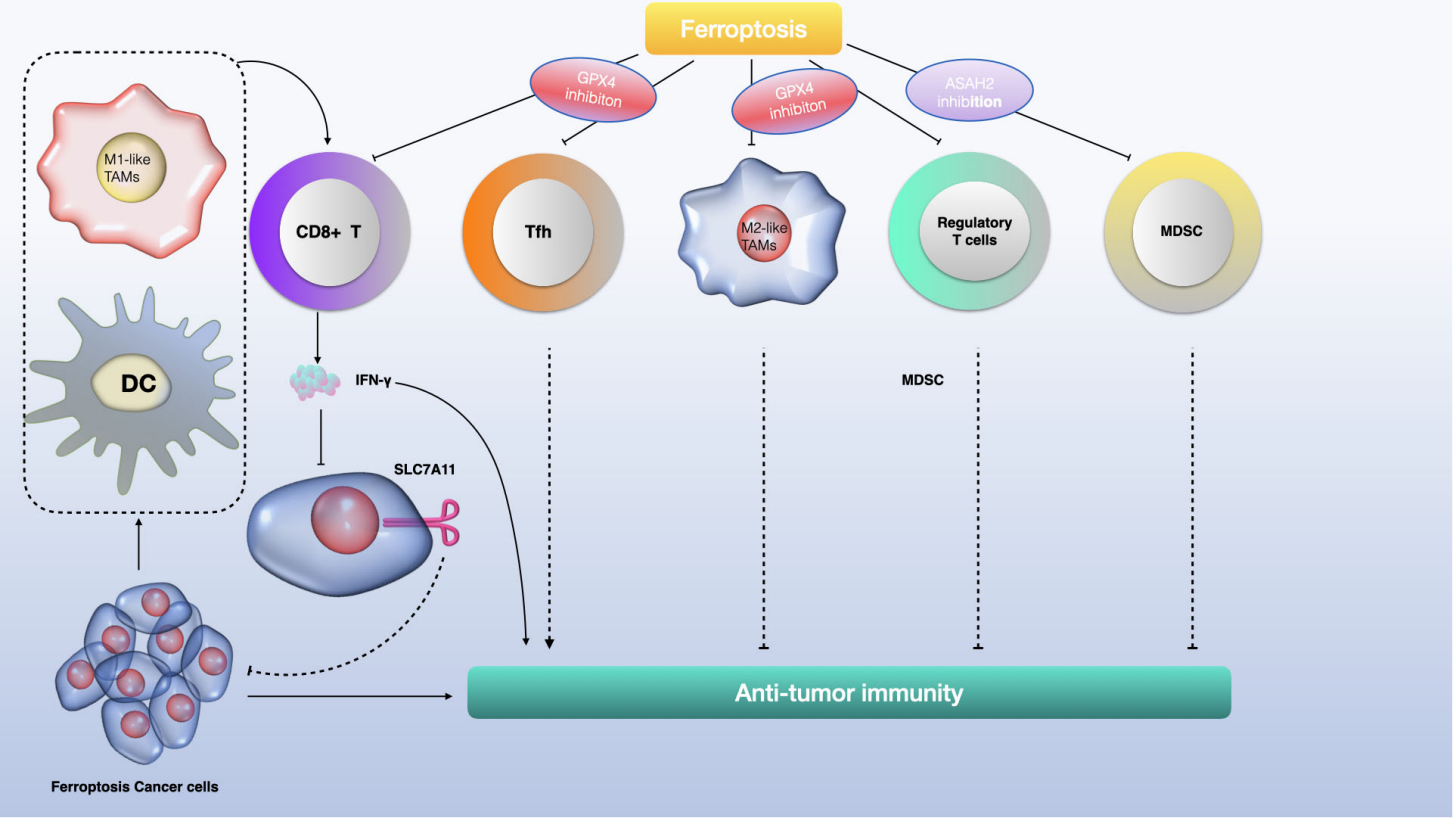
The role of ferroptosis in glioma immunity involves a complex interaction of immune cells and molecular pathways. CD8+ T cells produce interferon-gamma (IFNγ), activating the interferon receptor (INFR) which then suppresses SLC7A11 transcription through STAT1, promoting ferroptosis in tumor cells. Macrophages release TGFβ1, which via SMAD proteins, downregulates system xCT, leading to lipid ROS-induced ferroptosis through the glutathione (GSH) and glutathione peroxidase 4 (GPX4) axis. Ferroptotic glioma cells release damage-associated molecular patterns (DAMPs) like HMGB1 and arachidonic acid (AA), which attract and activate immune cells. Conversely, DAMPs like HMGB1, KRAS-G12D, and 8-hydroxyguanosine (8-OHG) can alter macrophage function in the tumor microenvironment. Notably, KRAS-G12D interaction with the AGER receptor on macrophages promotes M2 macrophage polarization, potentially hindering antitumor immunity.

Ferroptosis plays a critical role in the growth and treatment of gliomas, but its mechanisms require further exploration (145). Unlike most regulated cell death (RCD) effector molecules, which are typically proteases or porins such as caspases in apoptosis and mixed lineage kinase domain-like protein (MLKL) in necroptosis, the effector molecules in ferroptosis are less clear (146–148). Phospholipid hydroperoxides (PLOOH) are currently considered the primary executors of ferroptosis, yet it’s uncertain if there are additional effector molecules downstream of PLOOH (103, 149). The interaction between ferroptosis and other RCD forms is also not fully understood. Some aspects of ferroptosis, like lipid peroxidation and regulators including GPX4 and SLC7A11, are also involved in other RCD types. Ferroptosis may influence the tumor immune microenvironment (TIME), affecting glioma development and treatment (see **Figure 7**).

Currently, biomarkers like GPX4, ACSL4, P53, and FTH1 are used in diagnosing and treating gliomas, but they are not definitive standards (35, 150). Reliable biomarkers that can accurately predict tumor response to ferroptosis induction, especially those detectable in-patient blood, urine, feces, and tumor tissue, are urgently needed. It’s also unclear which glioma patients are more susceptible to ferroptosis treatments. Assessments combining iron levels, gene expression, and mutations might help identify patients who would benefit most from ferroptosis, such as those with gliomas overexpressing SLC7A11 being potential targets for SLC7A11 inhibitors.

Ferroptosis has a dual role in tumor development and treatment. While it promotes glioma cell death, it can also diminish the efficacy of glioma treatments by increasing Treg cells, neutrophils, and M2-polarized macrophages in the TIME, thus suppressing antitumor immunity (151). Tumor cells undergoing ferroptosis might induce stress in surrounding tumor cells, enabling them to avoid ferroptosis by secreting cytokines (149). The nature of substances released by tumor cells post-ferroptosis and their effects on the surrounding cells and TIME require further investigation (128, 152). More evidence is needed to confirm whether cytokines released after ferroptosis enable surrounding glioma cells to evade immune surveillance and regulate TIME (152, 153).

## Discussion

Targeting ferroptosis open up exciting avenues in cancer, but there are still unsolved questions in this field. Firstly, iron plays multiple roles in ferroptosis, and its function extends beyond redox reactions. A widely accepted model suggests iron is involved in generating lipid reactive oxygen species (ROS), either through Fenton chemistry or via iron-dependent oxidases (154). Iron’s necessity in ferroptosis might also reflect its role as a cofactor for various metabolic enzymes involved in ROS generation, such as the LOX family of enzymes or prolyl 4-hydroxylase isoform 1 (PHD1) (155, 156).

Secondly, the exact molecular executor of ferroptosis is not entirely clear. The oxidative fragmentation of polyunsaturated fatty acids (PUFAs) and resulting membrane lipid damage might be sufficient to induce cell death, possibly through plasma membrane permeabilization and damage to intracellular organelle membranes. Alternatively, the fragmented products of oxidized PUFAs, such as the toxic 4-hydroxynonenal (4-HNE), might promote death by reacting with and inactivating essential cellular proteins. The detoxification of 4-HNE by aldo-keto reductase family 1, member C (AKR1C), and its regulation by NRF2, indicates that 4-HNE accumulation may be a key ferroptotic driver (157, 158). However, the possibility of a specific death-inducing protein or protein complex activated downstream of lipid-ROS accumulation cannot be ruled out, warranting further research (159).

Thirdly, identifying molecular markers for cells undergoing ferroptosis remains challenging. Current methods mainly rely on observing increased cellular ROS and the effectiveness of ferroptosis inhibitors or iron chelators in preventing cell death. The mRNA expression of prostaglandin E synthase 2 (PTGS2) and ChaC glutathione-specific gamma-glutamylcyclotransferase 1 (CHAC1) has been found to elevate in cells undergoing ferroptosis (35, 159). However, these markers are not practical for use in live cells or intact tissues. An increase in heme oxygenase-1 (HO-1) expression upon erastin-mediated ferroptosis induction has been observed, but the universality of this marker across different cells and initiation pathways needs further validation (160). Thus, there is an ongoing search for more reliable ferroptosis markers for *in vivo* studies.

Fourthly, regarding the evolutionary aspect of ferroptosis, it poses an interesting question. Considering that iron-dependent oxidative metabolism has been a crucial part of life for billions of years, ferroptosis might indeed be one of the most ancient forms of programmed cell death. This suggests that evolutionary forces might have driven organisms to utilize this ROS/iron-driven cell death process for their own benefit, an intriguing area for further research and exploration.

In addition, recent studies have identified cuproptosis as a newly identified form of programmed cell death, which plays vital roles in tumorigenesis. Some researchers have shown that ferroptosis might interact with either cuproptosis or necroptosis. For example, it was demonstrated by Lifeng F. et al. that ferroptosis inducers enhance copper-induced cell death through depleting intracellular glutathione in liver cancer cells (161). While both ferroptosis and cuproptosis processes involve metal ions and contribute to cell death, their exact interrelationship is still under investigation. The distinct mechanisms suggest potential therapeutic targets in diseases where dysregulation of cell death is a factor. However, it’s important to note that research in these areas, especially cuproptosis, is still evolving, and the full extent of their relationship and implications in human health and disease are not yet fully understood.

Ferroptosis, as a novel mode of programmed cell death, is distinct from other types of regulated cell death due to its iron-dependent lipid peroxidation accumulation. This review highlights the regulatory mechanisms of ferroptosis and its multifaceted roles in the development and progression of glioma. Ferroptosis not only triggers the death of glioma cells but also influences their growth, invasion, migration, and resistance.

However, challenges such as poor blood-brain barrier (BBB) penetration and potential compensatory mechanisms limit the effectiveness of ferroptosis agents in glioma therapy. To address these challenges, it has been proposed that nanoengineered systems could improve the targeted delivery of drugs, thereby enhancing the effectiveness of glioma treatments. Despite the promising advantages of ferroptosis in treating glioma, there is a need for multidisciplinary collaboration to further investigate its benefits and drawbacks. Such research is crucial for evaluating the potential value of targeting ferroptosis in clinical applications.

## Author contributions

HW: Software, Writing – original draft. YL: Software, Supervision, Investigation, Writing – original draft. SC: Software, Writing – original draft. XL: Methodology, Writing – original draft. DT: Validation, Writing – review & editing. SL: Supervision, Writing – review & editing. HZ: Funding acquisition, Supervision, Writing – original draft, Writing – review & editing.
